# Non-small cell lung cancer associated microRNA expression signature: integrated bioinformatics analysis, validation and clinical significance

**DOI:** 10.18632/oncotarget.15596

**Published:** 2017-02-21

**Authors:** Chunyu Li, Yunhong Yin, Xiao Liu, Xuejiao Xi, Weixiao Xue, Yiqing Qu

**Affiliations:** ^1^ Department of Respiratory Medicine, Qilu Hospital of Shandong University, Jinan 250012, China

**Keywords:** NSCLC, microRNA, robust rank aggregation, integrative analysis, biomarker

## Abstract

Recently, increasing studies of miRNA expression profiling has confirmed that miRNA plays an essential role in non-small cell lung cancer (NSCLC). However, inconsistent or discrepant results exist in these researches. In present study, we performed an integrative analysis of 32 miRNA profiling studies compared the differentially expressed miRNA between NSCLC tissue and non-cancerous lung tissue to identify candidate miRNAs associated with NSCLC. 7 upregulated and 10 downregulated miRNAs were identified as miRNA integrated-signature using Robust Rank Aggregation (RRA) method. qRT-PCR demonstrated that miR-21-5p, miR-210, miR-205-5p, miR-182-5p, miR-31-5p, miR-183-5p and miR-96-5p were up-regulated, whereas miR-126-3p, miR-30a-5p, miR-451a, miR-143-3p and miR-30d-5p were down-regulated more than 2 folds in the NSCLC, which was further validated in Tumor Cancer Genome Atlas (TCGA) database. Receiver operating characteristic (ROC) curve analysis confirmed that 9 miRNAs had good predictive performance (AUC > 0.9). Cox regression analysis revealed that miR-21-5p (hazard ratio [HR]: 1.616, 95% CI: 1.114–2.342, *p* = 0.011) and miR-30d-5p (HR: 0.578, 95% CI: 0.400–0.835, *p* = 0.003) were independent prognostic factors in NSCLC for overall survival. The accumulative effects of the two miRNAs on the prognosis of NSCLC were further estimated. The results showed that patients with two positive markers had a worse prognosis than those with one or none positive marker. In conclusion, this study contributes to the comprehension of the role of miRNAs in NSCLC and provides a basis for further clinical application.

## INTRODUCTION

Lung cancer, mainly consisted of non-small cell lung cancer (NSCLC), is the most frequently diagnosed carcinoma and the leading cause of cancer-related death worldwide [1]. Despite it has a little improvement, the prognosis of lung cancer is still poor, with less than 18% of patients surviving more than 5 years, partly due to the fact that majority of cases are diagnosed at a late stage [2]. The survival of these patients relies remarkably on diagnosis. Therefore, identification of new biomarkers for the early diagnosis of NSCLC is crucial for the patients to receive optimal therapeutic regimen As early as possible.

MiRNAs, a novel class of short non-coding RNA molecules, play essential roles via posttranscriptional regulation of gene expression in almost all biological processes, including cell proliferation, differentiation, apoptosis, etc. Many miRNA profiling studies reveal the correlation between miRNAs and human diseases, especially cancers. Large numbers of differentially dysregulated miRNAs involved in cancer have been identified by high-throughput technologies across various normal and cancer tissues [3–5]. Thus, miRNAs have been regarded as potential biomarkers for diagnosis and precise predictions of prognosis for NSCLC, as well as targets for treatment [6, 7]. Although emerging evidences supported miRNAs as potential biomarkers of NSCLC, there were inconsistent or discrepant results among these researches due to the existence of various drawbacks, such as limited sample size, application of different profiling platforms, diverse methods for data collection and analysis, and increasing discovery of new miRNAs [8–10].

To overcome these limitations, we need an integrated and unbiased manner to analyze the results and obtain more significant miRNAs. Robust rank aggregation (RRA) approach [11], used for comparison of various ranked gene lists, is an accurate and effective method for identification of differentially expressed miRNA integrated-signature [12], in which a *P-value* would be assigned for all miRNAs in the ranked lists to re-rank these miRNAs and decide their significance.

Thus, we performed the integrated analysis to identify the key miRNA signatures as well as their molecular pathway in NSCLC patients, and then we validated the expression of these miRNA signatures using qRT-PCR and TCGA datasets. Obtained results might contribute to the diagnostics, therapeutics and prognosis for NSCLC patients.

## RESULTS

### Selection and characteristics of the datasets

**Figure 1:**
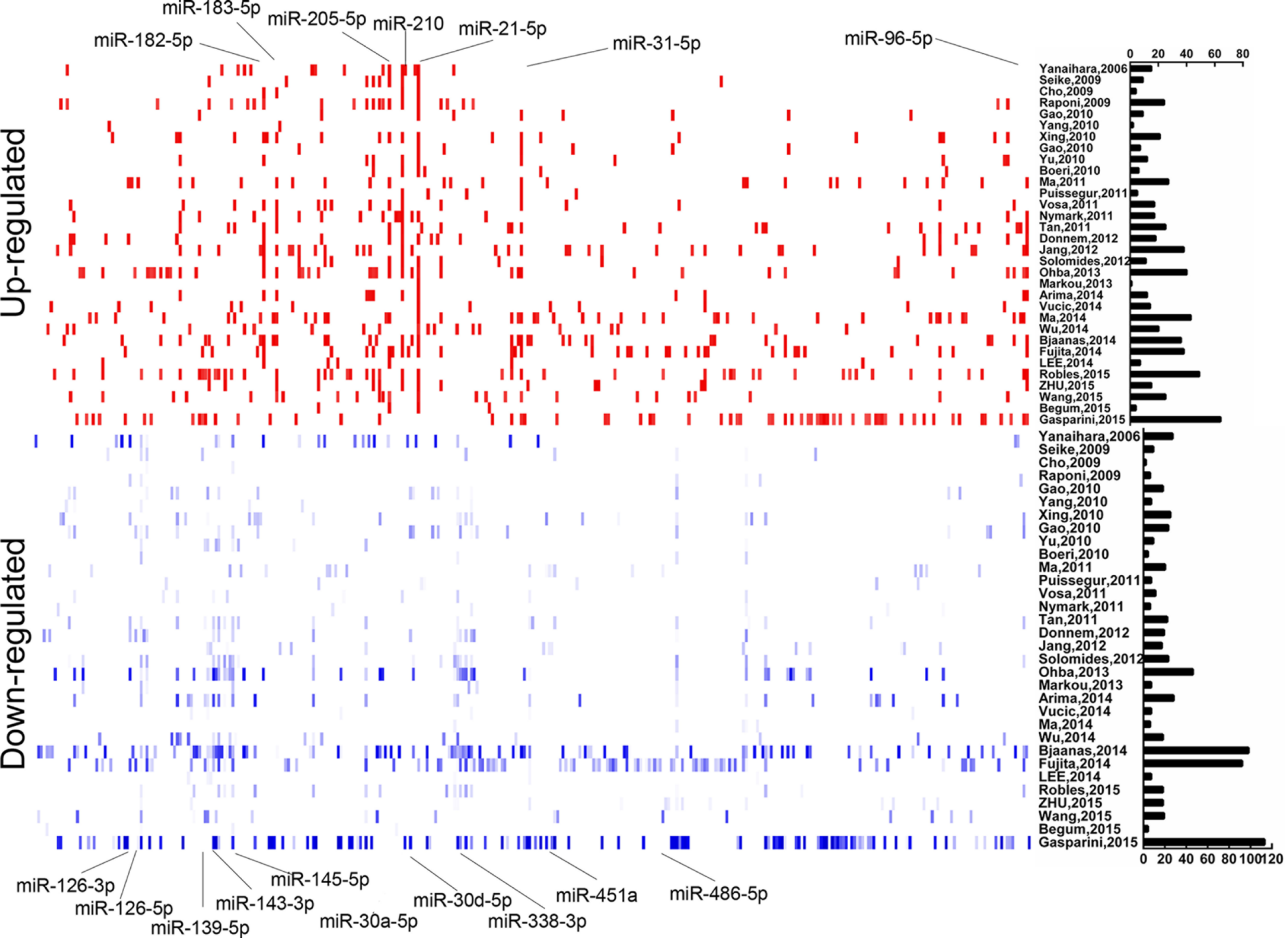
Distribution of NSCLC differentially dysregulated miRNAs extracted from 32 miRNA profiles studies Upregulated and downregulated miRNAs were shown as short red and blue vertical bars, respectively. The number of miRNAs in each study is graphically depicted on the right. The positions of NSCLC integrated-signature miRNAs were marked.

According to the inclusion criteria, 32 independent full-text studies were retrieved from public databases (Pubmed, GEO, and ArrayExpress), All these studies were published between 2006 and 2015. MiRNA sequencing technique was applied in 4 studies [8, 13–15] to identify the differentially expressed profiles, and the others used miRNA microarray chip technology. A simple summary of the studies was described in Table 1. In total, 1321 tumor and 930 non-cancerous samples were extracted in our study. The number of cases included ranged from 3 to 187 (median 20) over the studies. Diverse profiling platforms were applied and the median number of miRNAs detected was 688 (ranging from 235 to 2006) in these studies. Distribution of differentially dysregulated miRNA was shown in Figure 1. A total of 314 significantly up-expressed and 373 significantly down-expressed miRNAs were extracted from all studies. Moreover, 80 miRNAs with inconsistent alteration was found, suggesting that they were both up-regulated and down-regulated in different studies. The counts of remarkably alterative miRNAs varied extremely over these studies, but at least one up- and two down-regulated miRNAs was reported in each study.

**Table 1:** Characteristics of the studies

First author and reference	Region	Number of miRNA probes	Tumor type	Number of samples	Time
Yanaihara [10]	North America	352	SCC, AD	104 pairs	2006.3
Seike [16]	North America	389	SCC, AD	28 pairs	2009.6
Cho [17]	Asia	470	AD	10 pairs	2009.6
Raponi [18]	North America	328	SCC	61TU, 10 N	2009.7
Gao [19]	Asia	730	SCC, AD	8 pairs	2010.2
Yang [20]	Asia	711	SCC	3 pairs	2010.3
Xing [21]	North America	818	SCC	15 pairs	2010.6
Gao [22]	Asia	730	SCC	4 pairs	2010.6
Yu [23]	North America	377	AD	20 pairs	2010.12
Boeri [24]	North America	235	SCC, AD	24 pairs + 4 TU	2010.12
Ma [25]	Asia	858	SCC, AD	6 pairs	2011.1
Puissegur [26]	Europe	409	NSCLC	20 pairs	2011.3
Võsa [27]	Europe	858	SCC, AD	24 pairs + 14 TU + 3 N	2011.7
Nymark [28]	Europe	723	various	26 pairs	2011.8
Tan [29]	Asia	924	SCC	174 pairs + 13TU + 14N	2011.11
Donnem [9]	Europe	564	SCC, AD	30TU + 10N	2012.1
Jang [3]	North America	858	AD	56 pairs	2012.7
Solomides [30]	North America	817	SCC, AD	42 TU + 14N	2012.11
Ohba*	Asia	600	various	4 AD + 5 SCC + 8 N	2013.1
Markou [31]	Europe	320	SCC, AD	19 pairs	2013.5
Arima [4]	Asia	470	various	80 AD + 29 SCC + 5 N	2014.1
Vucic [15]	Europe	1372	AD	94 pairs	2014.1
Ma [8]	North America	896	SCC, AD	8 pairs	2014.1
Wu [32]	Asia	365	NSCLC	5 pairs	2014.2
Bjaanæs [33]	Europe	1205	AD	154 TU + 20 N	2014.3
Fujita [5]	Asia	1719	SCC, AD	29 pairs	2014.3
LEE [13]	Asia	1372	SCC, AD	9 pairs	2014.9
Robles [34]	North America	654	AD	32 TU + 30 N	2015.4
ZHU [35]	Asia	2006	SCC, AD	44 pairs	2015.5
Wang [14]	Asia	368	SCC	19 pairs	2015.5
Begum [36]	North America	688	SCC, AD	8 pairs	2015.8
Gasparini [7]	Europe	800	SCC, AD	67 TU + 18 N	2015.12

AD, adenocarcinoma; SCC, squamous cell carcinoma; TU, tumor samples; N, non-cancerous samples; pairs, TU and N samples from the same patient; ‘*’, without publication.

### NSCLC associated microRNA expression signature

Seven up-expressed (miR-21-5p, miR-210, miR-183-5p, miR-182-5p, miR-205-5p, miR-31-5p, miR-96-5p) and ten down-expressed (miR-126-3p, miR-30a-5p, miR-486-5p, miR-451a, miR-145-5p, miR-30d-5p, miR-143-3p, miR-139-5p, miR-126-5p, miR-338-3p) miRNAs were identified after integrated analysis by using RRA (Table 2). All signature miRNAs were revealed in at least 1/3 studies. Seven of the most significantly alterative miRNAs, miR-21-5p, miR-210, miR-31-5p, miR-126-3p, miR-30a-5p, miR-486-5p, miR-451a, were reported in 1/2 studies. The result of expression change for integrated-signature miRNAs was accordant among reported studies. For miR-126, two forms were exhibited with both its “major” (miR-126-3p) and “minor” (miR-126-5p). Majority of integrated-signature miRNAs belonged to the broadly conserved seed families. A cluster was defined as that miRNAs are located within the scope of 50 kb, are transcribed at the same direction and are not separated by a transcription unit or a miRNA in the opposite orientation. According to this criterion, 9 integrated-signature miRNAs belong to the cluster of two or more miRNAs.

**Table 2:** NSCLC associated microRNAs

microRNA	Chromosome	Permutation *p*-value	Corrected *p*-value	No. of Studies	Seed family	microRNA Cluster
**Upregulated**						
miR-21-5p	17q23.1	4.68E-29	9.38E-26	24	miR-21-5p/590-5p	-
miR-210	11p15.5	3.82E-26	7.66E-23	20	miR-210	-
miR-205-5p	1q32.2	1.12E-21	2.24E-18	15	miR-205-5p	-
miR-182-5p	7q32.2	9.38E-18	1.88E-14	16	miR-31-5p	-
miR-31-5p	9p21.3	6.72E-16	1.35E-12	14	miR-182-5p	miR-182/96/183
miR-183-5p	7q32.2	1.04E-13	2.09E-10	14	miR-183-5p	miR-182/96/183
miR-96-5p	7q32.2	1.28E-12	2.57E-09	12	miR-96-5p/1271-5p	miR-182/96/183
**Downregulated**						
miR-126-3p	9q34.3	4.30E-25	8.62E-22	21	miR-126-3p	-
miR-143-3p	6q13	8.24E-21	1.65E-17	19	miR-30abcdef-5p/384-5p	miR-30a/c-2
miR-451a	17q11.2	1.95E-20	3.91E-17	18	miR-451a	miR-4732/144/451
miR-486-5p	8p11.21	5.15E-19	1.03E-15	17	miR-486-5p	-
miR-145-5p	5q32	2.21E-12	4.44E-09	15	miR-145-5p/5195-3p	miR-143/145
miR-143-3p	5q32	3.02E-12	6.05E-09	14	miR-143-3p/4770/6088	miR-143/145
miR-30d-5p	8q24.22	8.41E-13	1.69E-09	13	miR-30abcdef/384-5p	miR-30b/d
miR-139-5p	11q13.4	1.21E-10	2.43E-07	11	miR-139-5p	-
miR-126-5p	9q34.3	3.60E-09	7.22E-06	11	miR-126-5p	-
miR-338-3p	17q25.3	1.18E-11	2.37E-08	11	miR-338-5p	miR-338/657/1250/3065

### Validation of expression of the integrated-signature miRNAs and clinical significance

**Figure 2:**
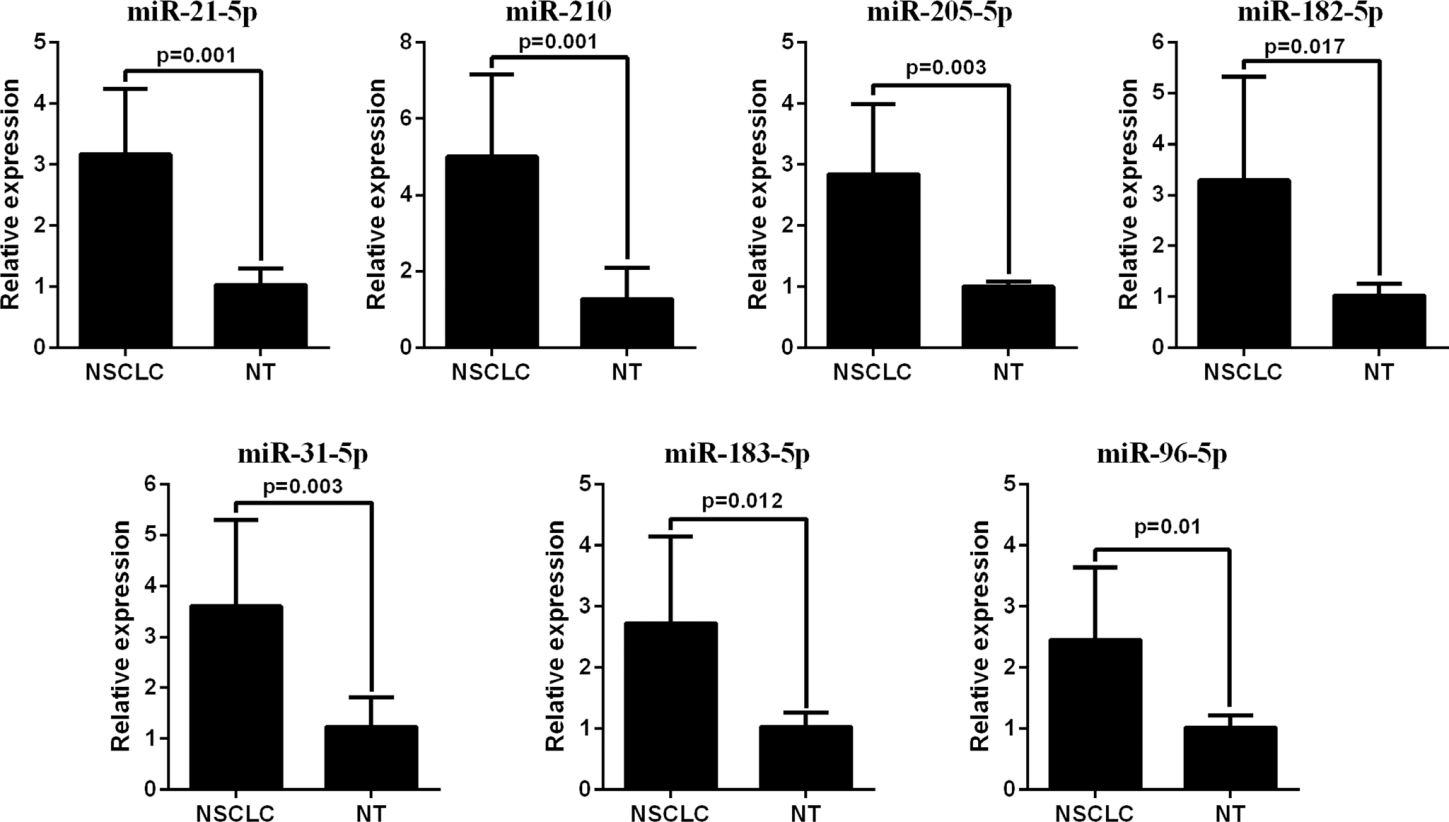
RT-PCR analysis of up-regulated miRNAs expression in the NSCLC tissues and the adjacent noncancerous lung tissues

**Figure 3:**
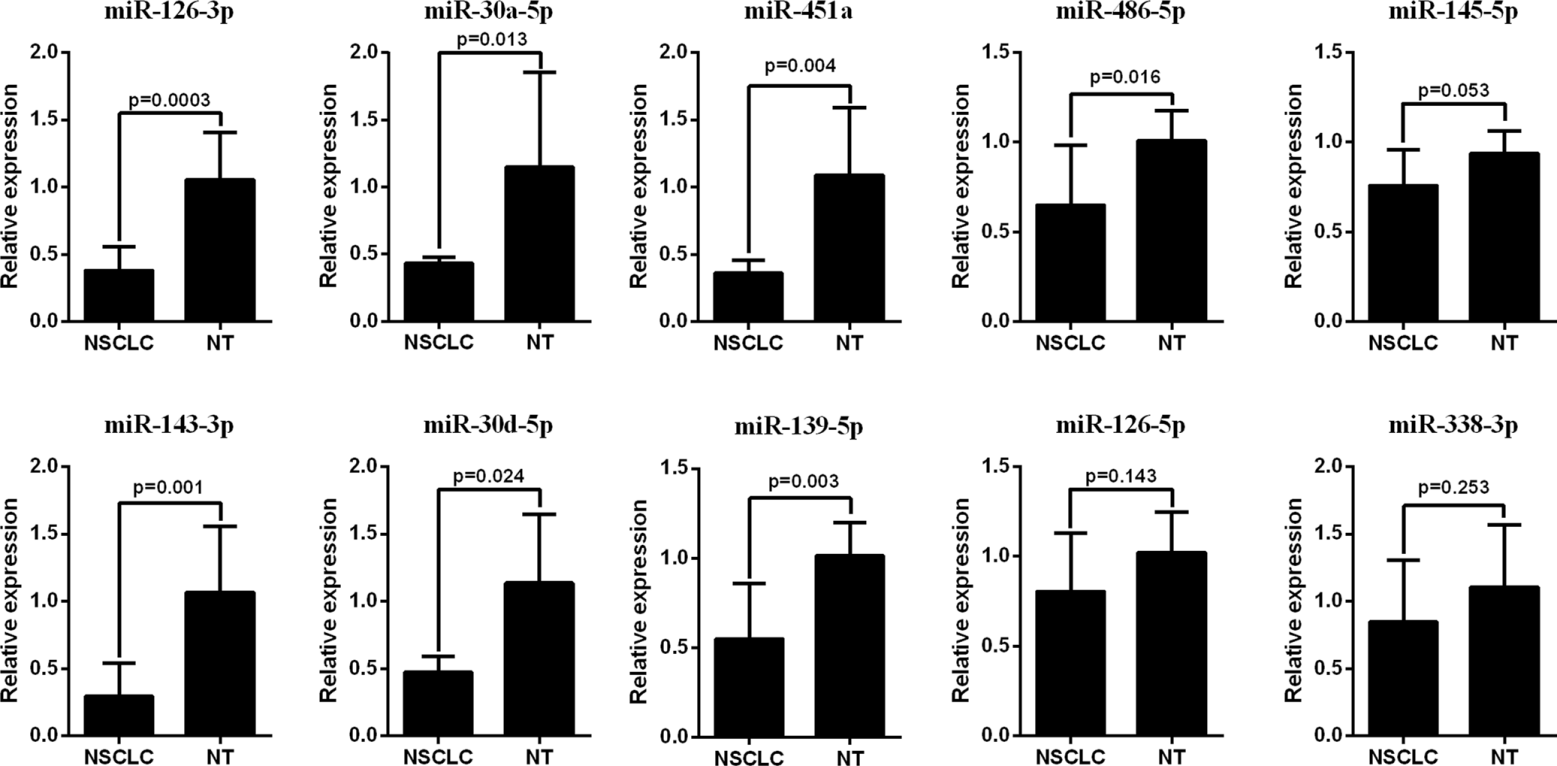
RT-PCR analysis of down-regulated miRNAs expression in the NSCLC tissues and the adjacent noncancerous lung tissues

**Figure 4:**
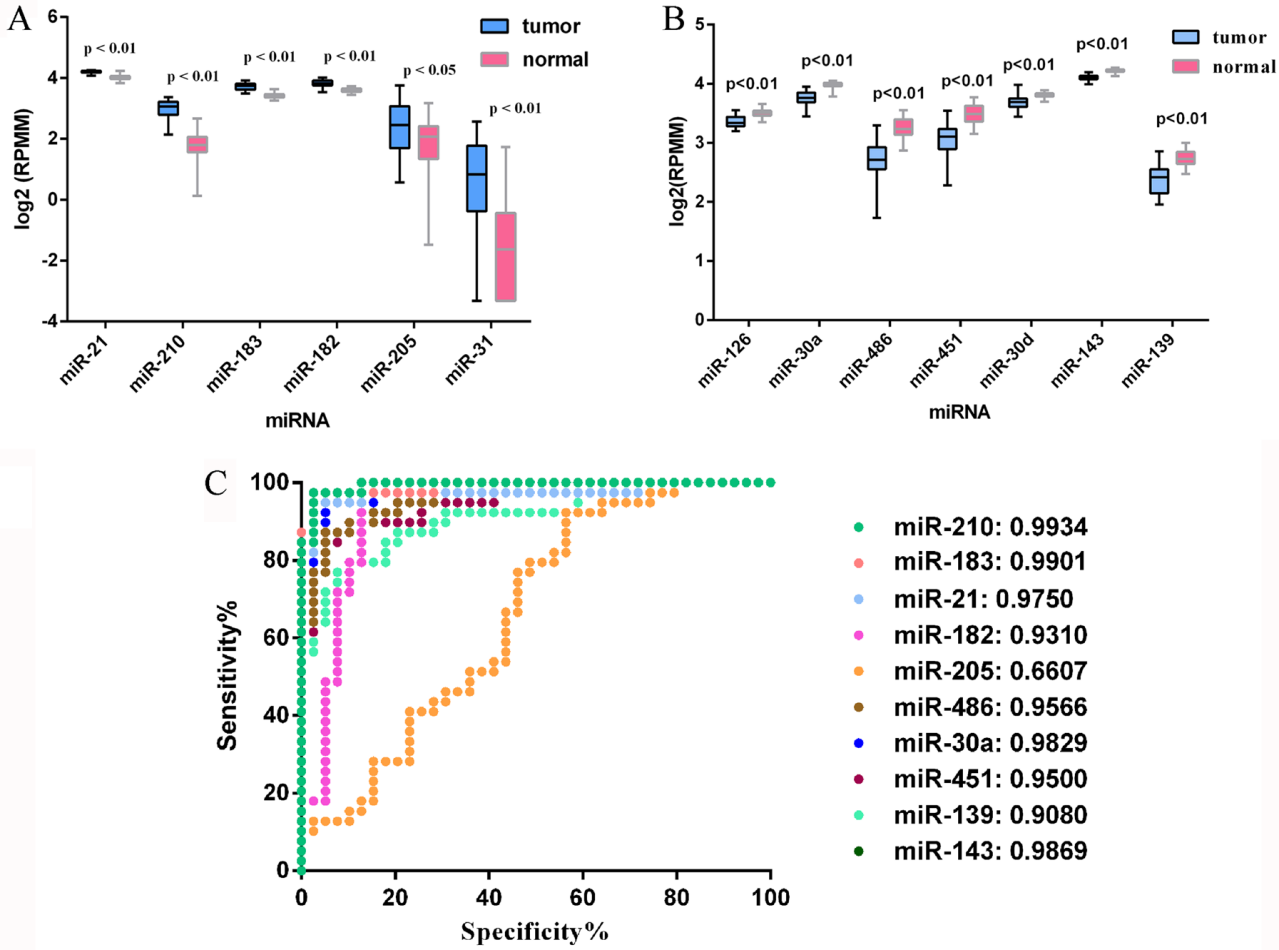
Validation of miRNAs expression in NSCLC on the TCGA dataset (**A**) Upregulated miRNAs expression. (**B**) Downregulated miRNAs expression. (**C**) ROC curve and AUC for performances of the miRNAs in NSCLC tissue classification. For boxplots, expression values of miRNAs were log2-transformed.

**Figure 5:**
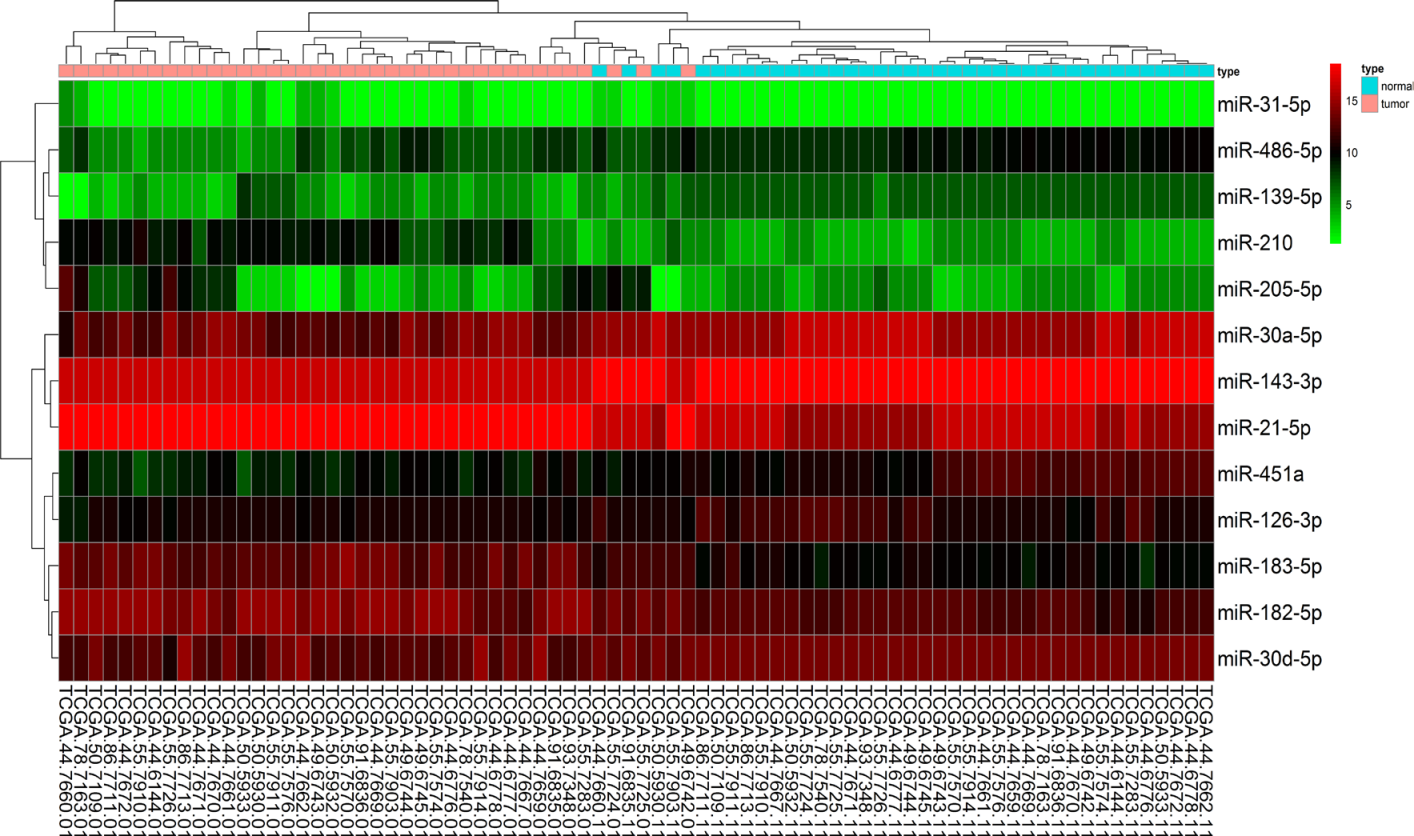
A heat map of differential expression of validated miRNAs for 39 pairs of lung adenocarcinoma and adjacent non-tumorous lung tissues in TCGA data base Red indicates high expression; black indicates moderate expression; green indicates low expression.

The qRT-PCR analysis was performed to validate the expression levels of the 17 integrated-signature miRNAs. The results showed that the expression of miR-21-5p, miR-210, miR-205-5p, miR-182-5p, miR-31-5p, miR-183-5p and miR-96-5p were up-regulated more than 2 folds (*P* < 0.05, Figure 2), whereas the expression of miR-126-3p, miR-30a-5p, miR-451a, miR-143-3p and miR-30d-5p were down-regulated more than 2 folds in the NSCLC tissues. Besides, the expression of miR-486-5p and miR-139-5p were also down-regulated, but failed to be more than 2 folds (*P* < 0.05, Figure 3). We further validated expression of the 17 miRNAs in TCGA data base (39 pairs of lung adenocarcinoma and adjacent non-tumorous lung tissues). Among the 17 most deregulated miRNAs, 13 miRNAs were confirmed to be significantly dysregulated, 3 miRNAs (miR-338-3p, miR-145-5p, miR-96-5p) failed to reach a statistical difference, 1 miRNA, miR-126-5p, was not listed (Figure 4A, Figure 4B). The expressions that changed more than 2-folds were found in miR-210, miR-183-5p, miR-21-5p, miR-182-5p, miR-205-5p, miR-486-5p, miR-30a-5p, miR-451a, miR-139-5p and miR-143-3p. A heat map of differential expression of validated miRNAs was shown in Figure 5. In addition, the miRNAs correlated to metastasis were assessed by using the TCGA data base (498 lung adenocarcinoma samples). The results showed that miR-145-5p, miR-451a, miR-21-5p were associated with distant metastasis, but only miR-21-5p had more than 1.5-fold changes. MiR-182-5p was associated with metastasis of lymph nodes, but the expressions changed less than 1.5-folds.

**Figure 6:**
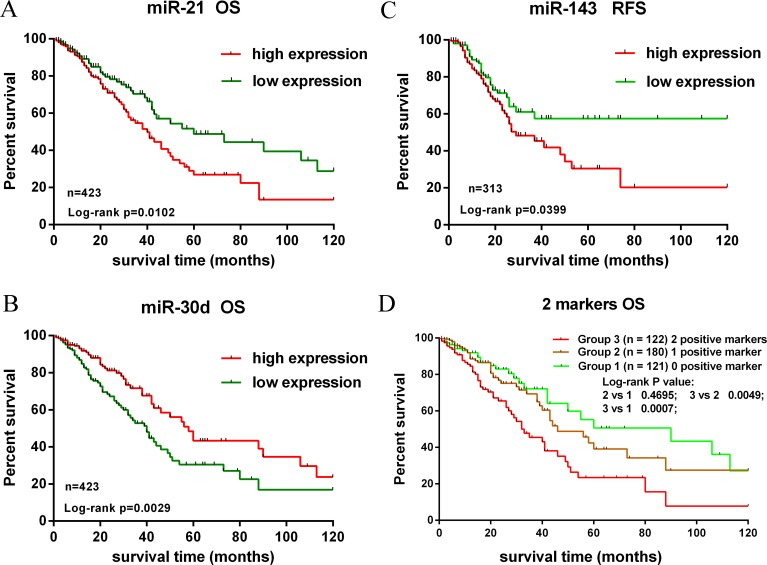
Kaplan-Meier survival analysis of overall survival and recurrence-free survival for validated miRNAs (**A**, **B**). The high expression of miR-21-5p and the low expression of miR-30d-5p were associated with poor prognosis for OS. (**C**) The high expression of miR-143-3p was associated with worse prognosis for RFS. (**D**) Kaplan-Meier analysis in patients with NSCLC according to the number of positive markers. The patients were divided into 3 Groups: 0 positive marker (group 1), 1 positive marker (group 2), 2 positive markers (Group 3). Patients of group 3 had a shorter OS.

The performances of these 10 validated miRNAs (fold change > 2) in NSCLC tissue classification were estimated using receiver operating characteristic (ROC) curve. Each miRNA had a good predictive performance and the Area under the ROC curve (AUC) was more than 0.9, with the exception of miR-205-5p, whose AUC was 0.6607 (Figure 4C). Furthermore, we constructed a prognostic classifier using Cox regression analysis in the TCGA data base (423 adenocarcinoma (AD) cases with overall survival and 313 adenocarcinoma cases with relapse-free survival). Of the 13 validated miRNAs by TCGA data base, only three miRNAs were selected: miR-21-5p (HR: 1.616, 95% CI: 1.114–2.342, *p* = 0.011) for overall survival (OS), miR-30d-5p (HR: 0.578, 95% CI: 0.400–0.835, *p* = 0.003) for OS, and miR-143-3p (HR: 1.575, 95% CI: 1.019–2.433, *p* = 0.041) for relapse-free survival (RFS), respectively. K-M survival analysis showed the three miRNAs could predict the clinical outcome (Figure 6A–6C). The findings indicated the high expression of miR-21-5p and the low expression of miR-30d-5p were all significant predictors of poor prognosis for OS in patients with NSCLC. To assess the integrated effects of the two miRNAs expression on the prognosis, we divided the 423 patients into three groups according to the number of positive markers from miR-21-5p high expression and miR-30d-5p low expression. Namely, group1 (none positive marker), group2 (one positive marker), group3 (two positive markers). Cox regression analysis and Kaplan-Meier survival analysis were executed and the differences between three groups were estimated. The results demonstrated that the patients with two positive markers (*n* = 122) had a worse OS than those with one positive marker (*n* = 180) (HR: 1.776, 95% CI 1.181–2.674, *p* = 0.006), than those with none positive marker (*n* = 121) (HR: 2.212, 95% CI 1.376–3.559, *p* = 0.001). However, the group2 failed to reveal a significantly statistical difference compared to group1 (*p* > 0.05) (Figure 6D).

### Targets prediction and functional enrichment

**Figure 7:**
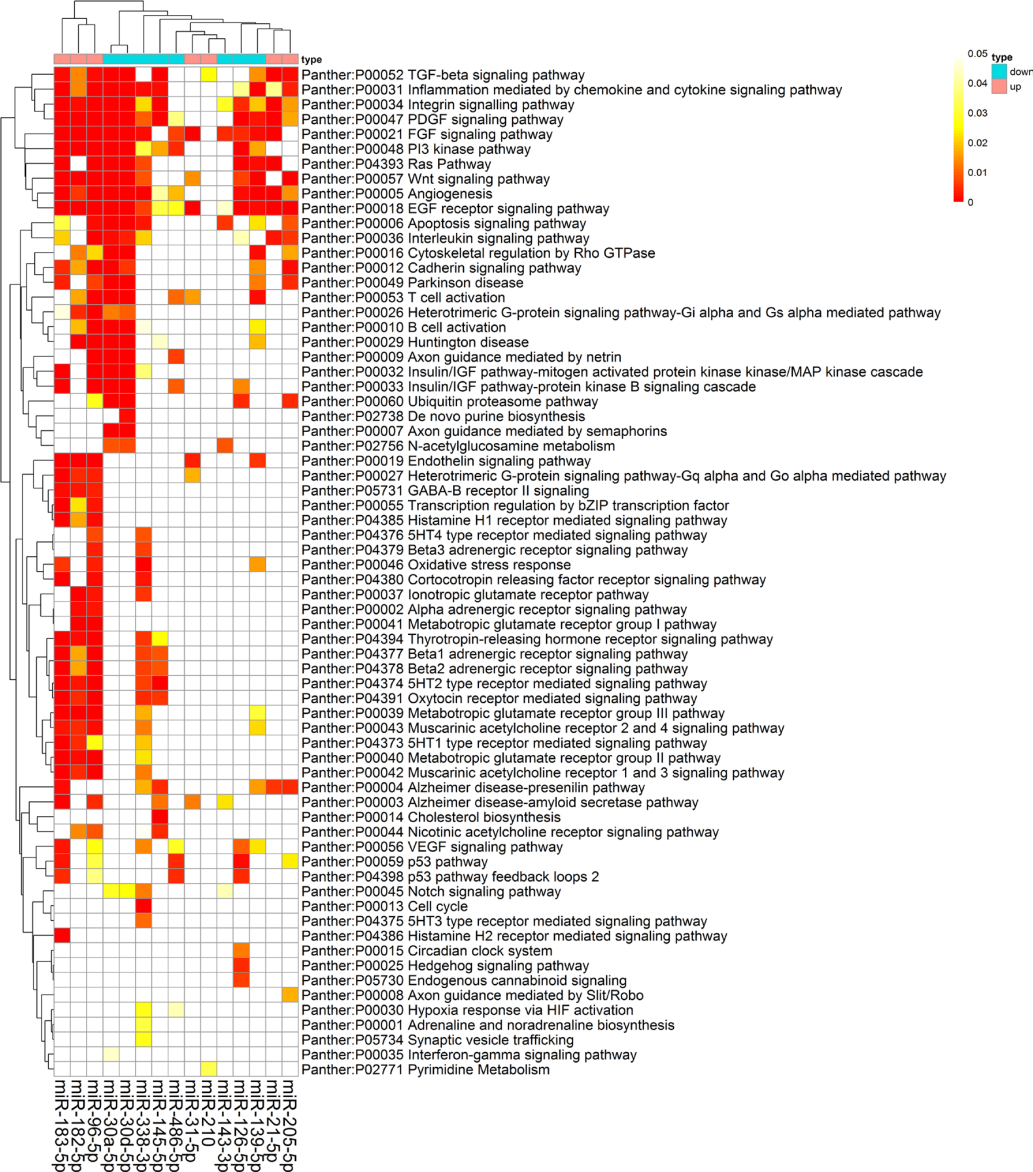
Panther pathway enrichment of targets by validated miRNAs The heat map was constructed using the validated targets and GeneCodis web tool, which showed the results of panther pathway enrichment analysis. The intensity of color represents the FDR-corrected *p-value*. Clustering was performed using Pearson correlation and average linkage method.

The high-precision target prediction for integrated-signature miRNAs was performed. Target genes were acquired from both prediction algorithms (TargetScan v7.1, miRDB and DIANA) and experimentally supported targets from TarBase and starBase. The results showed that miR-96-5p, miR-30a-5p and miR-30d-5p had the largest number of consensus target genes, whereas miR-126-3p, miR-210 and miR-451a had the smallest number of consensus targets. The counts of predicted targets were presented in Supplementary Figure 1. To elucidate the biological function of the miRNAs, we implemented enrichment analyses using consensus targets. As the result, 71 Panther pathways, 126 KEGG pathways, and 829 GO processes were enriched by the miRNAs targets. The most significantly enriched panther pathways concentrated on EGF receptor signaling pathway, integrin signaling pathway, PDGF signaling pathway, Wnt signaling pathway, FGF signaling pathway, angiogenesis, Ras pathway, and TGF-beta signaling pathway, the majority of which were reported to act as a crucial role in carcinogenesis (Figure 7). KEGG pathways enriched remarkably were mainly associated with characteristics of cancer, such as pathways in cancer, focal adhesion, MAPK signaling pathway, regulation of actin cytoskeleton, and neurotrophin signaling pathway. The GO processes that were markedly enriched mostly involved in regulation of transcription. Ten GO processes, KEGG pathways and Panther pathways most strongly enriched by consensus targets were shown in Table 3.

**Table 3:** The pathways and GO processes most strongly enriched by targets of validated microRNAs

Function enrichment	FDR	Targets
**GO processes**		
GO:0006355: regulation of transcription, DNA-dependent	1.44E-99	584
GO:0007165: signal transduction	3.90E-65	415
GO:0045944: positive regulation of transcription from RNA polymerase II promoter	1.73E-60	255
GO:0007275: multicellular organismal development	2.49E-57	344
GO:0045893: positive regulation of transcription, DNA-dependent	4.03E-53	213
GO:0045892: negative regulation of transcription, DNA-dependent	7.61E-50	189
GO:0000122: negative regulation of transcription from RNA polymerase II promoter	2.83E-46	188
GO:0007411: axon guidance	3.19E-46	156
GO:0007264: small GTPase mediated signal transduction	1.86E-41	151
GO:0007399: nervous system development	1.95E-41	179
**KEGG pathways**		
Kegg:05200: Pathways in cancer	6.81E-32	140
Kegg:04510: Focal adhesion	7.54E-30	100
Kegg:04010: MAPK signaling pathway	6.53E-30	119
Kegg:04360: Axon guidance	9.56E-30	77
Kegg:04810: Regulation of actin cytoskeleton	7.85E-27	99
Kegg:04722: Neurotrophin signaling pathway	5.66E-24	69
Kegg:04144: Endocytosis	3.31E-23	89
Kegg:04520: Adherens junction	5.84E-22	48
Kegg:04141: Protein processing in endoplasmic reticulum	1.07E-19	74
Kegg:04910: Insulin signaling pathway	9.19E-19	65
**Panther Pathways**		
Panther:P00018: EGF receptor signaling pathway	3.82E-21	63
Panther:P00034: Integrin signalling pathway	1.99E-20	75
Panther:P00047: PDGF signaling pathway	2.15E-17	62
Panther:P00057: Wnt signaling pathway	2.92E-17	102
Panther:P00021: FGF signaling pathway	1.16E-16	55
Panther:P00005: Angiogenesis	3.34E-16	67
Panther:P04393: Ras Pathway	3.89E-14	39
Panther:P00031: Inflammation mediated by chemokine and cytokine signaling pathway	5.79E-13	73
Panther:P00052: TGF-beta signaling pathway	5.03E-12	43
Panther:P00029: Huntington disease	1.34E-11	52

## DISCUSSION

MiRNAs have been identified as the potential biomarkers for diagnosis and clinical prognosis of many cancerous diseases. Although increasing miRNA profiling studies were performed to find the differentially dysregulated miRNAs, these efforts often presented inconsistent results across the different studies. Traditional meta-analysis has been done previously to determine differentially expressed miRNAs in cancer through a classical vote-counting method [37, 38]. However, such rigorous approach was often defective due to the existence of heterogeneity between different miRNA profiling platforms and the unavailability of raw data. In current study, to overcome these defects, an integrated analysis using RRA method was performed for identification of differentially dysregulated miRNAs from 32 independent profiling experiments. The miRNAs were re-ranked and their significances were re-determined by assigning a *P-value* for each miRNA. Finally, 7 upregulated and 10 downregulated integrated signature miRNAs were identified.

The majority of integrated-signature miRNAs were known to be functionally crucial in NSCLC. MiR-21 is upregulated and associated with poor prognosis in NSCLC [19, 31]. MiR-21 could promote tumorigenesis of NSCLC by potentiating indirectly the Ras/MEK/ERK pathway and inhibiting cell apoptosis [39]. Besides, it is shown to promote the growth and invasion of NSCLC through directly targeting PTEN [40]. Overexpression of miR-210 in NSCLC can decrease mitochondrial components related to cell survival and the activation of HIF-1 activity [26]. MiR-182-5p and miR-183-5p were found to be up-regulated in NSCLC tissue compared with adjacent normal lung tissue [17], and the miR-183∼96∼182 cluster could inhibit the invasion and metastasis of lung cancer through directly suppressing the expression of Foxf2 [41].

MiR-205 was identified as a potential accurate marker to distinguish SCC from other classifications of NSCLC [42]. It has also been shown to promote the growth, metastasis and chemoresistance of NSCLC cells by inhibiting the expression of PTEN [43]. MiR-31, one of oncogenic miRNAs, promotes lung cancer cell growth and tumorigenesis by suppressing specific tumor suppressors [44]. MiR-126-3p and miR-126-5p, two forms of miR-126, were associated with angiogenesis and cell proliferation in cancer [45], which were also down-regulation in present study. MiR-126 suppressed the expression of vascular endothelial growth factor A and potentiated the sensitivity of NSCLC cells to chemotherapy drugs [46]. MiR-30a-5p and miR-30d-5p had highest number of consensus targets in our study, which associated with the regulation of angiogenesis, apoptosis, invasion and metastasis. Consistent with our results, previous study has proved that miR-486-5p was significantly down-regulated in NSCLC tissues, and could inhibit cell proliferation, promote apoptosis, and hinder cell-cycle progression by targeting oncogene CDK4 [47]. Previous study had shown that miR-451a could enhance the sensitivity of NSCLC cells to cisplatin [48]. Furthermore, miR-451a could regulate survival of NSCLC cells through the inhibition of ras-related protein 14 (RAB14) and might to be a promising therapeutic target for NSCLC patients [49].

MiR-143 inhibited the genesis and development of NSCLC through directly suppressing the expression of its target genes [50]. MiR-145 is down-regulation in NSCLC and the restoration of miR-145 expression remarkably suppresses the growth of lung adenocarcinoma cells with EGFR mutation [17]. MiR-139-5p is associated with cancer cell proliferation, metastasis, and promotes apoptosis via the inhibition of c-Met [51]. MiR-338-3p inhibited the invasion and migration of NSCLC cells through the suppression of EMT-related transcription factor Sox4 and might serve as a potential therapeutic target for NSCLC [52]. Interestingly, many extensively investigated lung cancer-related miRNAs such as let-7, miR-34, miR-155 or miR-223 were not part of NSCLC integrated-miRNA signature. For example, miR-223 has been confirmed to play an essential role in development of NSCLC, but failed to reach a statistical significance in present study.

The expression of 17 integrated-signature miRNAs was validated using qRT-PCR and TCGA database. To assess the diagnostic value of these validated miRNAs in NSCLC tissue classification, we performed ROC curve analysis. The result showed that each miRNA had a good diagnostic performance, with the exception of miR-205-5p. Therefore, these miRNAs may serve as prospective biomarkers for the diagnosis of NSCLC. Further studies could be executed to evaluate the optimal diagnostic threshold value of these miRNAs in NSCLC through the use of more clinical data. In present study, we also estimated the performances of validated miRNAs to identify metastasis of NSCLC. The results demonstrated that miR-21-5p was associated with distant metastasis. Previous study has shown that inhibition of miR-21 expression was able to repress proliferation, invasion, and migration of A549 cells [53]. Thus, miR-21 may be useful as an effective indicator related to metastasis.

In addition, the Cox regression analysis showed that high expression of miR-21-5p and low expression of miR-30d-5p were independent hazard factors for shorter OS in NSCLC. Consistent with our findings, high expression of miR-21-5p as a perfect prognostic biomarker has been confirmed to be associated with worse OS both in NSCLC tissues and plasma [19, 31], respectively. Moreover, we reported that low expression of miR-30d-5p was associated with poor OS in NSCLC in the first time. Conversely, previous study showed that high expression of miR-30d in serum of NSCLC patients was associated with unfavorable survival [54]. A possible reason for the inconsistency was the use of different samples in both two studies. miR-30d-5p was down-regulation in NSCLC tissues in our integrated analysis (13 studies), but up-regulation in NSCLC serum samples compared to that in normal volunteers [55]. Further studies might be performed to elucidate the specific mechanisms.

The accumulative effect of the two miRNAs expression on the prognosis of NSCLC was further investigated. The result demonstrated that patients with 2 positive markers had a significantly worse prognosis (OS) compared with 1 or 0 positive marker. Therefore, the combination of the two markers may be better prognostic factor for shorter OS in NSCLC. Previous studies revealed that miR-143-3p was down-regulated in NSCLC tissue and suppressed metastasis of NSCLC cells. However, the Cox regression analysis and K-M survival analysis showed that high expression of miR-143-3p was positive correlation to worse RFS in NSCLC, consistent with the study in bladder cancer [56]. The possible reason was that Cox regression analysis only considered single factor of miRNA expression, and other factors (like age, TNM stages, treatment strategies, etc.) associated with survival were not included owing to the incomplete data in TCGA database. Multi-factor regression analysis based on more complete clinical data might be performed to illustrate the result in further studies.

The enrichment analysis of these miRNAs target genes suggested that the validated miRNAs were crucial regulator in the oncogenic process, which involved in several signaling pathways related to tumor genesis and progression. Therefore, these miRNAs might be also significant candidate biomarkers for identifying or monitoring relapse during postoperative follow-up in NSCLC. However, there are some limitations in our study. First, we did not perform validation experiments for biological function of these miRNAs and their targets. Functional analysis for the mechanism of these candidate miRNAs and potential targets in the progression of NSCLC should be researched in our future work. Second, considering miRNAs of carcinoma tissues are difficult to be detected, the serum miRNA level as biomarker for clinical diagnosis is more ideal. In addition, we believe that these miRNAs might be potential biomarkers for the early diagnosis of NSCLC patients and future studies should be performed in this issue using large, prospective and multi-center cohort.

In conclusion, 17 integrated-signature miRNAs were identified from 32 miRNA expression profiling studies. The expression of these miRNAs was validated using qRT-PCR test and TCGA database. The analyses of clinical significance showed that the majority of validated miRNAs were prospective biomarkers for diagnosis and predictions of prognosis for NSCLC. To gain sufficient sensitivity and specificity, the optimal diagnostic threshold value of the miRNAs should be determined in further clinical studies. In addition, targets prediction and functional enrichment analysis would contribute to the comprehension of the role of miRNAs in NSCLC. Further studies could focus on the underlying mechanisms of these miRNAs in genesis and progression of NSCLC and their clinical application.

## MATERIALS AND METHODS

### Studies inclusion and exclusion

MiRNA expression profiling studies for lung cancer, published prior to December 31st, 2015, were searched via Gene Expression Omnibus (GEO), ArrayExpress and Pubmed database. The search strategy was performed according to the term: (miR-* OR miRNA OR microRNA) AND (lung AND (tumor OR cancer OR carcinoma)) AND (profil* OR expression). Every study was estimated thoroughly through Full text. The included studies met the following criteria: 1. miRNA expression profiling studies (miRNA microarray chip technology or miRNA sequencing technique); 2. Primary experimental studies published in English language; 3. Studies compared miRNA expression between lung cancer and non-cancerous lung tissue in human. Expression studies using cell lines or body fluid (such as serum, saliva, peripheral blood, etc.) were excluded. Studies that only analyzed diverse histologic subtypes but did not include non-cancerous tissue were also excluded.

### Standardization of miRNA names

For these studies focused on only NSCLC (AD and/or SCC), the lists of differentially expressed miRNAs were extracted from corresponding studies. If the miRNAs lists were not available in the publications, the authors would be contacted directly for Supplemental Data. The studies that included multiple tumor subtypes were re-analyzed by using GEO2R tool or a limma package in R language between only NSCLC samples and non-cancerous lung tissue, other tumor subtypes were excluded. Studies without publication or obtained from GEO were also re-analyzed. The differentially dysregulated miRNAs were obtained according to the criterion: fold change > ± 1.5, FDR < 0.05. All miRNA names were standardized according to miRBase version 21 [57]. The nomenclature of miR/miR* were replaced with the -5p/−3p nomenclature, and many previous “major” miRNA names were re-designated according to miRBase database vesion21. Non-human miRNAs probes were eliminated from the studies. Pre-miRNAs were used in the analyses after the standardization, which were reported in some of the studies.

### Datasets construction

The extracted miRNAs were ranked on the basis of statistical test fold changes. A hierarchical cluster analysis was carried out to estimate the correlativity between the results of these studies. The rank matrixes of up-regulated and down-regulated miRNA lists were constructed separately. Moreover, the rank of miRNA from up-regulated and down-regulated miRNAs lists were both normalized, and a value was assigned to each miRNA. The value was one or 0.5 minus normalized rank of miRNA in upregulated or downregulated miRNA lists, separately. Value 0.5, above 0.5 or below 0.5 means that the miRNAs were not reported, upregulated or downregulated in corresponding study, separately.

### Statistical analysis

We used a new RRA method performed as an R package RobustRankAggreg to identify integrated miRNAs [11]. The leave one out cross-validation was executed in this method to estimate the stability of acquired *p*-values. The average *p-value*, representing the best *p-value* for each miRNAs, was acquired by repeating analysis 10,000 times. The integrated signature miRNAs selected in current study must reach statistical significance after Bonferroni correction and were reported by at least 1/3 studies.

### Quantitative real-time PCR of the integrated-signature miRNAs

To validate the expression of integrated miRNAs, 12 pairs of fresh NSCLC and adjacent noncancerous lung tissue samples were obtained from the Qilu Hospital of Shandong University between January and June, 2015. Written informed consent was obtained from all patients or their guardians. The use of all patient samples was approved by the Ethical Committee of the Qilu Hospital of Shandong University. All patients didn't undergo any chemotherapy or radiation therapy prior to operation and had no other malignancies accompanied. Clinical information of these patients was described in the Supplementary Table 1. Total RNA was extracted from these samples using TRIzol reagent (Life Technologies), following the manufacturer's instructions. The synthesis of first-strand cDNA contained two procedures. Firstly, the poly (A) tailing was synthesized with a Poly (A) Polymerase (RiboBio). Secondly, cDNA was synthesized using miDETECT A TrackTM Uni-RT Primer (RiboBio), Reverse Transcriptase mix (RiboBio) and the product of poly (A) tailing. Real-time PCR was performed with SYBR Premix Ex Taq TM (TakaRa) on RT-PCR system (ABI Prism 7000) according to the following protocol: 95°C for 5 min, followed by 35 cycles of 95°C for 10 sec, 60°C for 35 sec and then 95°C for 15 sec, 60°C for 1 min and 95°C for 15 sec, with addition of a cycle for every 0.5°C. The primers of these miRNAs and U6 were obtained from RiboBio Corporation (Guangzhou, China). The information of the primers was listed in Supplementary Table 2. The fold change of each miRNA expression was calculated using the ΔΔCT method.

### Validation of the integrated-signature miRNAs and clinical significance using TCGA database

To validated the expression of integrated-signature miRNAs, 39 pairs of lung adenocarcinoma and adjacent non-tumorous lung tissues were obtained from the TCGA data base (LUAD miRNA expression (IlluminaHiseq), *n* = 498). MiRNA expression information and corresponding clinical data for NSCLC were downloaded from the Cancer Browser (https://genome-cancer.ucsc.edu/). This study met the publication guidelines provided by TCGA. A limma package of R language was used to identify the differentially expressed miRNAs between lung adenocarcinoma and adjacent non-tumorous lung tissues. Corresponding heat map of miRNAs expression was constructed using pheatmap package of R language. Furthermore, the expression level of each miRNA was log2-transformed for further analysis. ROC curve was applied to estimate the predicted performances of the validated miRNAs for NSCLC tissue classification. The Cox regression analysis and Kaplan-Meier method were used to estimate the prognostic significance of the miRNAs for OS and RFS, and survival curves were compared through the log-rank test. The median value of miRNA expression was defined as cut-off value between high expression and low expression. The statistical analyses were carried out by using the SPSS 18.0. Statistical significance was defined as *p* < 0.05.

### MiRNA target prediction

The presumptive targets of miRNAs were predicted through three different algorithms: TargetScan v7.1 (http://www.targetscan.org/) [58], DIANAmicroT-CDS Web Server v5.0 (http://www.microrna.gr/microT-CDS) [59] and miRDB (http://www.mirdb.org/miRDB/) [60]. DIANA target predictions were executed with miTG score threshold 0.7. Validated targets were obtained from TarBase v7.0 [61] and starBase v2.0 [62]. In conclusion, consensus targets were defined as genes predicted by at least 2 algorithms plus validated targets from starBase and TarBase.

### Enrichment analysis

To conduct enrichment analyses, the GeneCodis web tool (http://genecodis.dacya.ucm.es/) [63] was used for Panther and KEGG pathways and GO processes analyses. Predicted targets of all integrated miRNAs were used as input for enrichment analyses. The pathways or GO processes that the number of supportive genes was more than four were selected. A visualized heat map was constructed through False discovery rate (FDR)-corrected *p*-values. Pearson correlation and average linkage were applied to Cluster analysis of the heat map.

## SUPPLEMENTARY MATERIALS FIGURES AND TABLES



## Abbreviations

NSCLC: non-small cell lung cancer
AD: adenocarcinoma
SCC: squamous cell carcinoma
miRNA: microRNA
RRA: robust rank aggregation
TCGA: Tumor Cancer Genome Atlas
HR: hazard ratio
FDR: false discovery rate
qRT-PCR: quantitative real-time polymerase chain reaction
OS: overall survival
RFS: recurrence-free survival
ROC: receiver operating characteristic
AUC: Area under the ROC curve
