# Supplementary material for: Non-small cell lung cancer associated microRNA expression signature: integrated bioinformatics analysis, validation and clinical significance
Source: Oncotarget. 2017 Feb 21;8(15):24564–78. doi: 10.18632/oncotarget.15596 (PMC5421870; doi:10.18632/oncotarget.15596)
Supplement: Supplementary file 1 [file oncotarget-08-24564-s001.pdf]

Non-small cell lung cancer associated microRNA expression signature: integrated bioinformatics analysis, validation and clinical significance

Supplementary Materials

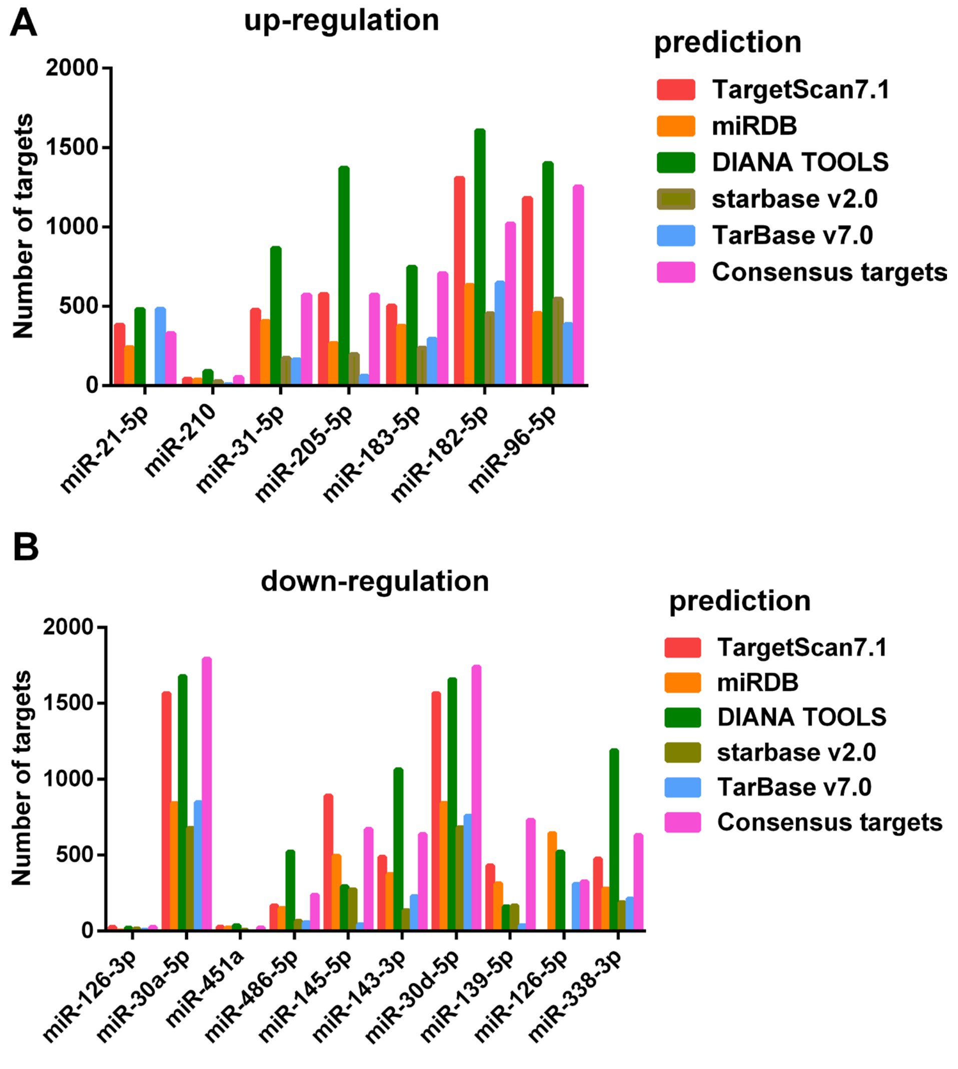

Supplementary Figure 1: Predicted and experimentally validated targets of integrated miRNAs. Shown is the number of targets predicted by three target prediction algorithms and validated target databases Tarbase and starBase. Consensus targets were defined as genes predicted by at least 2 algorithms plus validated targets from TarBase and starBase.

Supplementary Table 1: Characteristics of the patients

No.	gender	age	smoking history	histological type	tumor tissue site	pathologic stage
1	male	60	YES	AD	R-Middle	Stage IIA
2	male	70	YES	SCC	L-Lower	Stage IIA
3	female	51	NO	AD	R-Lower	Stage IA
4	male	78	NO	AD	R-Middle	Stage IIIA
5	male	73	YES	SCC	R-Upper	Stage IB
6	male	61	YES	AD	R-Upper	Stage IIB
7	male	59	YES	SCC	L-Upper	Stage IB
8	male	60	YES	SCC	L-Upper	Stage IIB
9	female	43	NO	AD	R-Lower	Stage IIA
10	female	63	NO	AD	R-Upper	Stage IIIA
11	male	36	YES	AD	R-Upper	Stage IIA
12	female	66	NO	AD	L-Upper	Stage IA

AD, adenocarcinoma; SCC, Squamous cell carcinoma.

Supplementary Table 2: Information of the primers

miRNA ID	Species	Accession number	Sequence
miR-21-5p	human	MIMAT0000076	uagcuuaucagacugauguuga
miR-210-3p	human	MIMAT0000267	cugugcgugugacagcggcuga
miR-205-5p	human	MIMAT0000266	uccuucauuccaccggagucug
miR-182-5p	human	MIMAT0000259	uuuggcaaugguagaacucacacu
miR-31-5p	human	MIMAT0000089	aggcaagaugcuggcauagcu
miR-183-5p	human	MIMAT0000261	uauggcacugguagaauucacu
miR-96-5p	human	MIMAT0000095	uuuggcacuagcacauuuuugcu
miR-126-3p	human	MIMAT0000445	ucguaccgugaguaauaaugcg
miR-30a-5p	human	MIMAT0000087	uguaaacauccucgacuggaag
miR-451a	human	MIMAT0001631	aaaccguuaccauuacugaguu
miR-486-5p	human	MIMAT0002177	uccuguacugagcugccccgag
miR-145-5p	human	MIMAT0000437	guccaguuuucccaggaaucccu
miR-143-3p	human	MIMAT0000435	ugagaugaagcacuguagcuc
miR-30d-5p	human	MIMAT0000245	uguaaacauccccgacuggaag
miR-139-5p	human	MIMAT0000250	ucuacagugcacgugucuccagu
miR-126-5p	human	MIMAT0000444	cauuauuacuuuugguacgcg
miR-338-3p	human	MIMAT0000763	uccagcaucagugauuuuguug
